# Acute Interstitial Nephritis and Oxalate Nephropathy After Rapid Pasireotide Response in Treatment-resistant Acromegaly

**DOI:** 10.1210/jcemcr/luae071

**Published:** 2024-05-20

**Authors:** Annabelle G Hayes, Mark J Penny, Karina Aivazian, Jerry R Greenfield

**Affiliations:** Department of Diabetes and Endocrinology, St. Vincent's Hospital, Sydney, NSW 2010, Australia; School of Clinical Medicine, St. Vincent's Campus, Faculty of Medicine and Health, University of New South Wales, Sydney, NSW 2010, Australia; Department of Renal Medicine and Transplantation, St. Vincent's Hospital, Darlinghurst, NSW 2010, Australia; School of Medicine, University of Notre Dame, Sydney, NSW 2010, Australia; Department of Tissue Pathology and Diagnostic Oncology, St. Vincent's Hospital and NSW Health Pathology, Sydney, NSW 2010, Australia; Faculty of Medicine and Health, The University of Sydney, Sydney, NSW 2050, Australia; Department of Diabetes and Endocrinology, St. Vincent's Hospital, Sydney, NSW 2010, Australia; School of Clinical Medicine, St. Vincent's Campus, Faculty of Medicine and Health, University of New South Wales, Sydney, NSW 2010, Australia; Clinical Diabetes, Appetite and Metabolism Laboratory, Garvan Institute of Medical Research, Sydney, NSW 2010, Australia

**Keywords:** oxalate nephropathy, pasireotide, exocrine insufficiency, acromegaly, interstitial nephritis

## Abstract

We report a case of interstitial nephritis, likely secondary to oxalate nephropathy, due to the development of pancreatic exocrine dysfunction after commencement of pasireotide for acromegaly. Pasireotide is known to impair insulin secretion but can also impair pancreatic exocrine function, hypothezised to result from high-affinity binding of somatostatin receptors 1, 2, 3, and 5. This has been an advantage in postoperative tissue anastomoses after pancreatic surgery, but exocrine insufficiency has not been reported when used for the treatment of acromegaly. A 73-year-old woman, diagnosed with acromegaly, was unable to achieve biochemical control despite 2 surgical resections of an invasive mammosomatotroph pituitary tumor and treatment with cabergoline and maximal-dose lanreotide. The tumor expressed somatostatin receptor type 5 but not somatostatin receptor type 2, predicting good response from pasireotide, which was commenced at 40 mg every 4 weeks. IGF-1 rapidly normalized, but the patient presented with nausea, anorexia, and acute kidney injury. Renal biopsy revealed acute-on-chronic interstitial nephritis, with numerous oxalate crystals. Increased fecal fat globules were noted on fat stain (3+), supporting malabsorption as an etiology of secondary enteric hyperoxaluria. Renal function recovered to near baseline over months following pasireotide withdrawal and high-dose glucocorticoids.

## Introduction

Pasireotide is a second-generation, pan-somatostatin analog, with higher receptor affinity, broader action, and longer half-life than octreotide and lanreotide with the exception of somatostatin receptor type 2 (SSTR2). While it is known to impair insulin secretion, it also results in pancreatic exocrine dysfunction, hypothesized to result from high-affinity binding of somatostatin receptors 1, 2, 3, and 5 (1, 2). This has been an advantage in postoperative tissue anastomoses after pancreatic surgery. We report a case of interstitial nephritis, likely secondary to oxalate nephropathy (ON) after commencing pasireotide.

## Case Presentation

A 73-year-old woman was diagnosed with acromegaly in 2020 in the setting of newly diagnosed type 2 diabetes after a Bentall's procedure. While glycated hemoglobin was modestly elevated at 6.0%, she was noted to have subtle acromegalic facies, including new prognathism. Further history revealed change in ring size, hypertension, obstructive sleep apnoea, and colonic polyps. Initial biochemical assessment included an IGF-1 threefold the upper limit of normal [75.9 nmol/L (580 ng/mL) normal reference range (RR) 6-27 nmol/L; 53-191 ng/mL] and a random GH of over double the upper limit of normal [25 mIU/L (8.3 µg/L) RR 0-10 mIU/L; 0-3.3 µg/L] (Table 1). Magnetic resonance imaging demonstrated a 2.4 × 2.5 × 3.8 cm pituitary mass, which extended superiorly into the suprasellar space and inferiorly, invading the clivus.

**Table 1.** Summary of key biochemical investigations through clinical course

	On diagnosis of Acromegaly	Prior to pasireotide	After 3 doses of pasireotide	Reference range
Adrenocorticotropic hormone (ACTH)	3.6 pmol/L (16.3 pg/mL)	-	< 1.5 pmol/L (< 6.8 pg/mL)	< 12 pmol/L (<54 pg/mL)
Cortisol (nmol/L)	396 nmol/L (14.6 µg/dL)	452 nmol/L (16.4 µg/dL)	376 nmol/L (13.6 µg/dL)	150-520 nmol/L (5.4-18.9 µg/dL)
Thyroid stimulating hormone (TSH)	0.18 mIU/L (0.18 µIU/mL)	0.78 mIU/L (0.78 µIU/mL)	2.40 mIU/L (2.40 µIU/mL)	0.40-4.80 mIU/L (0.40-4.80 µIU/mL)
Free thyroxine (FT4)	10.1 pmol/L (0.78 ng/dL)	10.5 pmol/L (0.82 ng/dL)	9.1 pmol/L (0.71 ng/dL)	8.0-16 pmol/L (0.62-1.24 ng/dL)
Growth Hormone (GH) (mIU/L)	25 mIU/L (8.3 µg/L)	4.6 mIU/L (1.5 µg/L)	1.7 mIU/L (0.57 µg/L)	0-10 mIU/L (0–3.3 µg/L)
Insulin-like Growth Factor-1 (IGF-1)	75.9 nmol/L (580 ng/mL)	65 nmol/L (497 ng/mL)	7.0 nmol/L (53 ng/mL)	7-25 nmol/L (53-191 ng/mL)
Prolactin	800 mIU/L (37.6 µg/dL)	1353 mIU/L (77.8 µg/dL)	155 mIU/L (7.3 µg/dL)	50-300 mIU/L (2.4-14.1 µg/dL)
Glucose	7.8 mmol/L (140 mg/dL)	5.2 mmol/L (94 mg/dL)	7.0 mmol/L (126 mg/dL)	3.6-6.0 mmol/L (65-108 mg/dL)
Urea	5.0 mmol/L (30.0 mg/dL)	9.2 mmol/L (55.3 mg/dL)	22.5 mmol/L (135.1 mg/dL)	4.0-9.0 mmol/L (24.0-54.1 mg/dL)
Creatinine	50 µmol/L (0.57 mg/dL)	95 µmol/L (1.07 mg/dL)	425 µmol/L (4.81 mg/dL)	45-90 µmol/L (0.51-1.02 mg/dL)
Estimated glomerular filtration rate (eGFR)	>90 mL/min/1.73m^2^	50 mL/min/1.73m^2^	8 L/min/1.73m^2^	>60 mL/min/1.73m^2^

**Figure 1.**
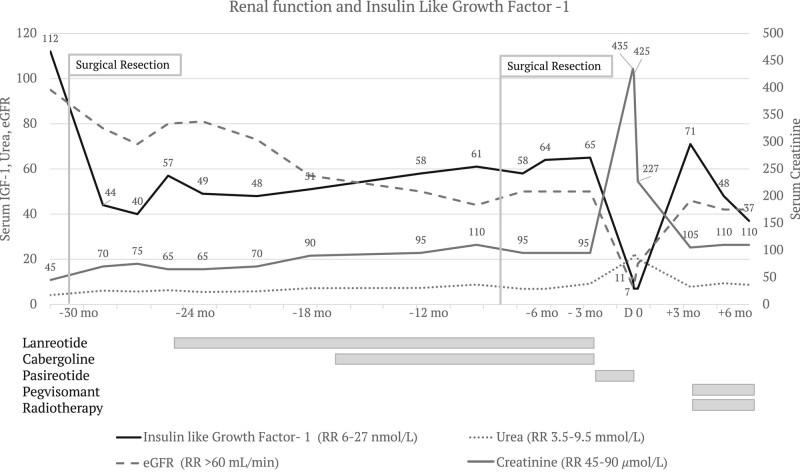
Renal function and IGF-1 over treatment course. IGF-1 was 112 nmol/L (857 ng/mL) (RR 6-27 nmol/L; 53-191 ng/mL) after diagnosis, prior to first surgical resection of the pituitary tumor. At the time creatinine and urea were at their lowest likely due to hyperfiltration in a state of GH and IGF-1 excess. Over the following 12 to 18 months IGF-1 was never normalized despite escalation of medical therapy with lanreotide, cabergoline, and a second surgical debulking. During this time, serum creatinine and urea demonstrated a slow deterioration after an initial expected rise after improvement in gross GH/IGF-1 excess. Pasireotide was effective at normalizing IGF-1, but treatment was complicated by acute-on-chronic interstitial nephritis with serum creatinine peaking at 435 μmol/L (RR 45-95). After pasireotide withdrawal, IGF-1 rose to 71 nmol/L (543 ng/dL) but has since responded to pegvisomant while radiotherapy takes effect.

Initial surgical debulking of an invasive, densely granulated mammosomatotroph pituitary tumor improved serum IGF-1 levels, but normalization was not achieved despite repeat surgical resection and treatment with cabergoline and maximal-dose lanreotide (Fig. 1). Immunohistochemistry was diffusely positive for both prolactin and GH, transcription factor PIT-1. Ki67 was increased at 5.3%, while mitotic count was not elevated (1 per 10 high-powered fields) clinicopathological grade per Trouillas was increased at 2B. Magnetic resonance imaging demonstrated residual tumor infiltration of the clivus from the level of the right petrous internal carotid artery to the level of the atlanto-occipital articulation. A positron emission tomography/computed tomography scan performed after administration of 154MBq of Ga 68 DOTATATE demonstrated faint avidity involving most of the clivus (SUVmax 3.3), suspicious for disease involvement but far lower than is typical for somatotroph tumors. There was no DOTATATE-avid distant disease.

The tumor expressed SSTR5 but not SSTR2, predicting good response from pasireotide, which was commenced at 40 mg every 4 weeks. IGF-1 rapidly normalized. However, after 3 doses the patient developed progressive nausea and lethargy.

## Diagnostic Assessment

The patient was noted to have an acute kidney injury (serum creatinine 425 µmol/L, baseline 100 µmol/L, RR 45-90 µmol/L). She was advised to present to the hospital. A fourth dose of pasireotide was not administered.

Serum cortisol was 13.6 µg/dL (374 nmol/L) (RR 5.4-18.9 µg/dL; 150-520 nmol/L). There was low-level proteinuria but no hematuria, glycosuria, casts, crystals, or evidence of infection. An autoimmune glomerulonephritis screen was negative, with normal antinuclear antibody, antineutrophilic cytoplasmic antibody, extractable nuclear antigen, double-stranded deoxyribonucleic acid, C-reactive protein, complement component 3, and complement component 4. Serum lipase was 160 U/L (RR 0-60 U/L), and amylase was also raised at 133 U/L (RR 0-100 U/L).

Renal imaging with a noncontrast computed tomography abdomen and pelvis excluded renal tract calculus and obstruction. The right kidney was noted to have a 13 mm benign hyperdense cyst at the upper pole, while the left kidney, spleen, pancreas, and left adrenal were normal.

**Figure 2.**
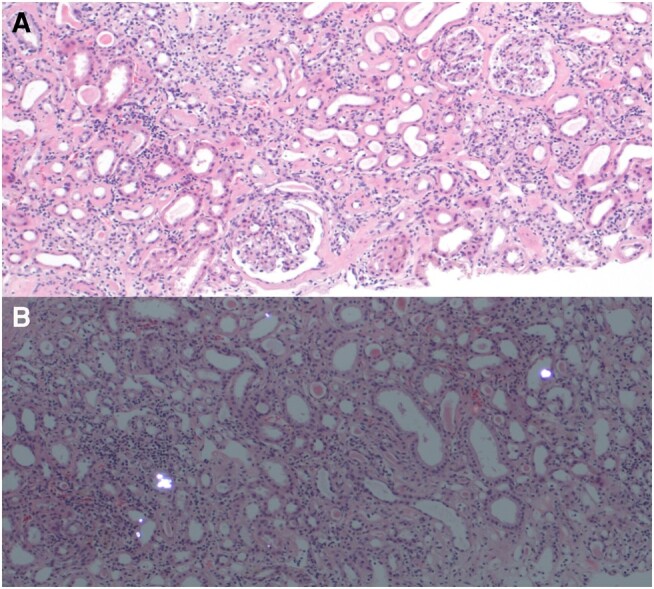
Renal biopsy. (A) Hematoxylin and eosin stain revealed acute-on-chronic interstitial nephritis, with global sclerosis in 4 of 24 glomeruli, mild to moderate interstitial fibrosis, and a conspicuous lymphocytic infiltrate, including the areas of the cortex not affected by fibrosis. (B) Numerous oxalate crystals were seen under polarized light.

Subsequent renal biopsy revealed global sclerosis in 4 of 24 glomeruli, mild to moderate interstitial fibrosis, and a conspicuous lymphocytic infiltrate, including the areas of the cortex not affected by fibrosis (Fig. 2). Tubular morphology was variable, with some areas of ectasia and atrophy and others of tubulitis. Calcium oxalate crystals were present in several tubules. Immunofluorescence did not show convincing evidence of immune deposits in tubular basement membranes. No arteriolosclerosis was seen, although subcortical fibrosis and obsolescent glomeruli were present, typically seen in long-term hypertension. Overall, the pattern of acute-on-chronic interstitial nephritis with numerous oxalate crystals raised the possibility of secondary hyperoxaluria contributing to interstitial injury.

Urine oxalate excretion, assayed 1 week after drug withdrawal, intravenous rehydration, and methylprednisolone, was high-normal (0.498 mmol/day, RR <0.500). Exocrine insufficiency was further suggested by increased fecal fat globules (3+) using the Sudan 111 stain. Fecal elastase level (Diasorin Liaison XL chemiluminescent immunoassay), assayed 1 week after initial presentation (>800 ug/g, RR >200 ug/g).

## Treatment

Pasireotide was ceased, last given prior to hospital presentation. The patient was treated for acute drug-induced interstitial nephritis with pulsed methylprednisolone and intensive intravenous hydration with crystalloid fluid. The patient continued on a slow wean of prednisolone starting at 37.5 mg after discharge. Although she never consumed high levels of dietary oxalate, further education was provided to ensure a low oxalate diet. She had no history of bariatric surgery, high vitamin C intake, or other established triggers of hyperoxalatemia.

## Outcome and Follow-up

The patient noted resolution in her symptoms on discharge from the hospital. Serum creatinine continued to improve returning to baseline (105 umol/L, RR 45-90 umol/L) over several months (Fig. 1). A repeat fecal fat stain was negative on follow-up.

The patient has commenced stereotactic radiotherapy as the residual mammosomatotroph tumor in the clivus was deemed surgically unresectable following multidisciplinary team review. IGF-1 rebounded to 71 nmol/L (543 ng/mL) (RR 7-25 nmol/L; 53-191 ng/mL) following pasireotide withdrawal. However, the patient has responded to pegvisomant treatment, falling to 48 nmol/L (367 ng/mL) within 1 month and 37 nmol/L at 3 months, while creatinine remains stable at 110 umol/L (Fig. 1).

## Discussion

ON is present in 0.2% to 4% of renal biopsies and is associated with acute tubular injury and acute or chronic interstitial nephritis (3, 4). Secondary ON is largely due to enteric hyperoxaluria, which occurs due to fat malabsorption. Excessive intestinal fat prevents calcium from binding oxalate in the intestine, leaving oxalate free for absorption (3). Malabsorptive bariatric surgery and pancreatic insufficiency account for most cases of secondary ON (3). Recent antibiotic use is also associated with up to a third of cases (3).

Histological appearance in this case closely reflects that of clinicopathological series of ON described in the literature (4). Of nearly 30 000 renal biopsies, of which 3000 were performed for acute kidney injury, 68 cases were confirmed to have secondary ON. All cases demonstrated oxalate crystals in the cortex, 88% also with medullary deposition, and all with acute tubular injury with interstitial inflammatory infiltrate. Like this case, 70% were found to have global glomerulosclerosis affecting a median 13% of sampled glomeruli (4).

There has been 1 case of ON previously described with first-generation somatostatin receptor analogue octreotide (5). However, the etiology was confounded by concurrent and prolonged antibiotic use for a multiresistant organism, which could feasibly explain hyperoxalatemia through depletion of *Oxalobacter formigenes*-mediated oxalate degradation in the gut (3).

Somatostatin, in addition to atropine, is a potent inhibitor of cholecystokinin (CCK)-mediated release of pancreatic enzyme secretion. CCK usually acts via the CCK receptor on pancreatic acinar cells, intrapancreatic neurons, and afferent cholinergic neurons (6).

Pasireotide is a pansomatostain receptor analog with a 33-fold higher affinity for SSTR5 than lanreotide (7). This explains the particular sensitivity in serum IGF-1 response in this patient, where the tumor expressed SSTR5 but not SSTR2. Pasireotide also has higher affinity for other somatostatin receptors than lanreotide, 65x higher for SSTR3, and 220x higher for SSTR1, which may further explain off-target effects of the small intestine and biliary system (7).

Pasireotide has been has been shown to reduce pancreatic acinar cell secretion using in vitro and in vivo models (8). This prompted use in patients undergoing pancreatectomy, where perioperative pasireotide was associated with a relative risk of 0.44 (95% confident interval 0.24-0.78) in reducing postoperative leak, fistula, or abscess as compared to placebo (9). In meta-analysis this was suggested but did not reach statistical significance (10).

An interesting feature of this case is the normal fecal elastase, conflicting with other measures suggesting exocrine pancreas insufficiency including fecal fat. Increased fecal fat is generally not apparent until lipase release is reduced to 10% of usual functioning (11). Acknowledging the low false-negative rates of the chemiluminescent fecal elastase assay (1.1%), the most likely explanation is the 1-week delay between cessation of pasireotide, treatment of renal impairment, and sampling until after treatment, thus missing the window acinar cell inhibition (11). Another possible explanation for the robust fecal elastase concentration is acinar cell hyperresponsiveness after inhibition and the release of preformed enzymes.

Similarly, urine oxalate levels remained high-normal when measured after several days of treatment including intravenous rehydration, lowering urinary oxalate concentrations (3). Oxalate clearance may have been reduced due to concurrent renal injury. Plasma oxalate measurement is not available at our center but could have been informative (3).

Background chronic tubulointerstitial changes on renal biopsy in this case were reflected in mild elevation in serum creatinine, which had been gradually up-trending since diagnosis of acromegaly 3 years prior. It is possible structural damage directly from GH/IGF-1 preceded the rise in serum creatinine, initially masked by GH-mediated hyperfiltration until initial tumor resection (Fig. 1) (12). However, direct drug toxicity remains a possibility.

ON generally carries a poor prognosis, depending on its etiology. After malaborptive bariatric surgery, progression to renal replacement therapies is approximately 50% (4). In this case, cessation of pasireotide and recovery of pancreatic exocrine function has reduced this risk significantly and allowed improvement in renal function as evidenced by a serum creatinine now returned to the patient's baseline.

## Learning Points

- Pasireotide binds with high affinity to somatostatin receptors 1, 2, 3, and 5, which can result in pancreatic endocrine and exocrine insufficiency.
- Oxalate nephropathy in the absence of a high oxalate diet should prompt consideration of pancreatic exocrine insufficiency.
- Hyperoxaluria should be considered in patients who develop renal dysfunction in the setting of pasireotide therapy.
- Key investigations of pancreatic exocrine insufficiency include fecal fat stain and fecal elastase.

## Data Availability

Original data generated and analyzed during this study are included in this published article.

## Abbreviations

CCK: cholecystokinin
ON: oxalate nephropathy
RR: normal reference range
SSTR: somatostatin receptor
